# Gender differences in the right atrial appendage and right atrium-related structures in patients with atrial fibrillation and their association with ablation outcomes

**DOI:** 10.1016/j.ijcha.2026.101945

**Published:** 2026-05-25

**Authors:** Tong Pan, Yang Liu, Cai-Ying Li, Shi-Bo Dong, Dan Zhang, Xiao-Nan Han

**Affiliations:** a Department of Radiology, Hebei General Hospital, Shijiazhuang, Hebei Province, China; b Department of Radiology, The Second Hospital of Hebei Medical University, Shijiazhuang, Hebei Province, China; c Department of Radiology, The Third Hospital of Hebei Medical University, Shijiazhuang, Hebei Province, China; d School of Medical Imaging, Hebei Medical University, Shijiazhuang, Hebei Province, China

## Abstract

**Objective:**

To quantitatively measure gender-specific anatomical structures of the right atrial appendage (RAA) and right atrium (RA) in atrial fibrillation (AF) patients, and explore their clinical significance for post-radiofrequency ablation (RFA) recurrence using 256-slice spiral Computed Tomography (CT).

**Method:**

This study included 321 AF patients (184 males and 137 females) undergoing RFA for the first time. All patients underwent a 256-slice spiral CT examination before surgery. The volume of the RA, RAA, and left atrium (LA); RAA height; short diameter, long diameter, area, and circumference of RAA base; RA anteroposterior diameter, tricuspid annulus diameter, crista terminalis, and cavo-tricuspid isthmus (CTI) were measured, and clinical data were collected.

**Results:**

After body surface area normalization, the RA volume and RA anteroposterior diameter in female patients with AF showed more significant structural remodeling than those in male patients. Both male and female patients with recurrent AF showed larger RAA and RA structural parameters. In females, short diameter of RAA base, crista terminalis thickness and persistent AF were independent recurrence predictors post-RFA, while short diameter of RAA base and AF duration were independent predictors in males (all *P* < 0.05). ROC analysis showed that short diameter of RAA base served as the strongest predictor of AF recurrence in both genders. The optimal cutoff values were 26.95 mm for females (sensitivity = 0.696, specificity = 0.824, AUC = 0.811, P = 0.000) and 25.55 mm for males (sensitivity = 0.700, specificity = 0.746, AUC = 0.786, P = 0.000).

**Conclusion:**

The short diameter of the RAA base had the highest predictive value for postoperative recurrence in both male and female patients.

## 1 Introduction

Atrial fibrillation (AF) is the most common arrhythmia, affecting 1%–2% of the global population. [1] Similar to many cardiovascular diseases, the occurrence, progression, and prognosis of AF are characterized by gender differences. The incidence of AF is higher in males than in females [2], while female AF patients present with more prominent symptoms, as well as an elevated risk of recurrence and mortality [3]. Studies on cardiac magnetic resonance (CMR) have revealed that female AF patients bear a heavier burden of atrial fibrosis [4], which increases the risk of recurrence after radiofrequency ablation (RFA). However the potential pathophysiological mechanism underlying this gender difference remains unclear. Some researchers have employed high-precision mapping techniques to investigate gender-related differences in the left atrial (LA) substrate, demonstrating a higher proportion of low LA voltage in female [5].

Currently, there is no consensus on the impact of gender on the outcomes of RFA for AF. Furthermore, some studies have indicated no significant difference in the recurrence after RFA [1]. To date, anatomical reconstruction of the right atrial appendage (RAA) and right atrium (RA) in AF patients of different genders has not been reported, nor has their relationship with postoperative recurrence.

Thus, the aim of our study is to quantitatively measure the morphological structures and related parameters of RAA and RA in AF patients of different genders, and to compare their structural differences. Additionally, we explored the application value of RAA and RA morphological features in predicting AF recurrence after RFA across genders.

## 2 Materials and methods

### 2.1 Study design and participants

From January 2020 to December 2020, 321 patients with AF who underwent RFA in our hospital were examined by Coronary Computed Tomographic Angiography (CCTA). All patients were diagnosed through electrocardiogram (ECG) and physical examination. AF recurrence after RFA was defined as rapid atrial arrhythmia (atrial tachycardia, atrial flutter, or AF) lasting > 30 s, recorded by ECG during the 3-month postoperative blanking period. Recurrence was evaluated at 3, 6, and 12 months postoperatively via telephone follow-up, outpatient surface ECG, and 24-hour ambulatory ECG, with a minimum 1-year follow-up. All patients signed informed consent before the CCTA examination. Inclusion criteria: (1) first successful RFA for AF; (2) available preoperative 256-slice spiral CT examination; (3) no severe postoperative complications causing death or loss of follow-up, including cardiac tamponade, atrial esophageal fistula, intraoperative acute myocardial infarction, intraoperative or postoperative stroke, and pericardial hemorrhage [6]. Exclusion criteria:(1) poor CCTA image quality precluding measurement of RAA volume, RA volume, and crista terminalis thickness; (2) CCTA contraindications (e.g., contrast agent allergy), pacemaker implantation, or other cardiac implants; (3) patients with obvious organic heart disease, such as valvular disease and congenital heart disease, as factors affecting RAA and RA dilatation may interfere with AF. All methods of this study were carried out in accordance with relevant guidelines and regulations. The study was approved by our hospital’s Institutional Ethics Committee (No. 2018-R245). The institutional review committee waived the requirement for informed consent owing to the retrospective nature of the study.

Ablation was performed under general anesthesia with the CARTO 3.0 three-dimensional (3D) mapping system. The subclavian or femoral vein was punctured to position a coronary sinus electrode, Subsequently, atrial septal puncture was performed to establish 3D reconstruction of the LA pulmonary vein. Linear mapping along the LA–pulmonary vein junction was completed, and bilateral pulmonary vein isolation with linear ablation was implemented under an artificial intelligence-guided high-power ablation protocol. Post-procedural electrophysiological stimulation was used to confirm the achievement of bidirectional conduction block between the pulmonary veins and the LA [7].

### 2.2 CCTA techniques and image post-processing & measurement

CCTA examination was performed in patients with AF before ablation using Phillips 256-slice CT (Brilliance iCT, Philips Healthcare, Amsterdam, Netherlands) in our hospital. Breath-holding training was performed before scanning. The scanning range was 0.5 cm below the tracheal bifurcation to the cardiac diaphragm. Iohexol contrast agent 350 mg/ml, 0.8 ml/kg was injected into the median cubital vein at a flow rate of 4–5 ml/s using a high-pressure syringe. Scanning parameters were as follows: tube voltage 80–120 kV, collimation 128 × 0.625 mm, pitch 0.18, matrix 512 × 512, tube current 280–350 mAs/r, and gantry rotation time 330 ms.

The Philips EBW 4.6 workstation was used to reconstruct 75% of the cardiac cycle, and 3D reconstruction of the RAA and RA was performed by volume rendering (VR) and multiplanar reconstruction (MPR). For anatomical demarcation of the RAA and its RAA base, the junction between the superior vena cava (SVC) and RA was delineated as the SVC orifice on axial images. Axial imaging demonstrated that the RAA is positioned superior to the SVC orifice and manifests as a saccular structure protruding anteriorly from the RA. The RAA base was perpendicular to the long axis of the RAA and was located at the level of the superior vena cava opening on axial images [8].

**Fig. 1:**
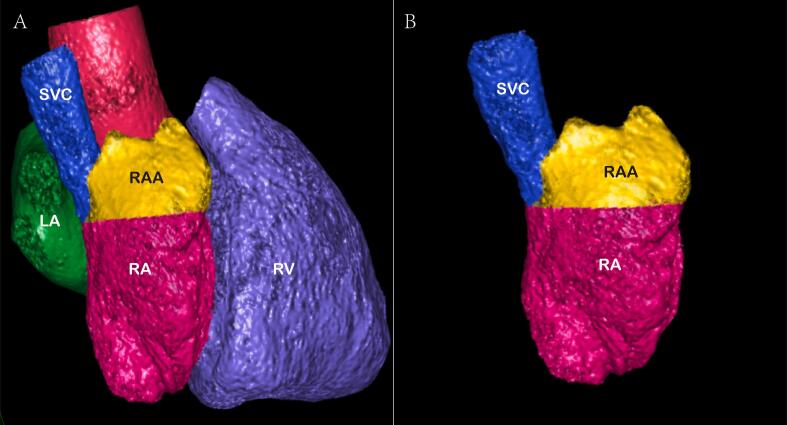
Three-dimensional image of the right atrial appendage (RAA), right atrial (RA),Left atrial (LA) volume (Fig. 1A-B).

**Fig. 2:**
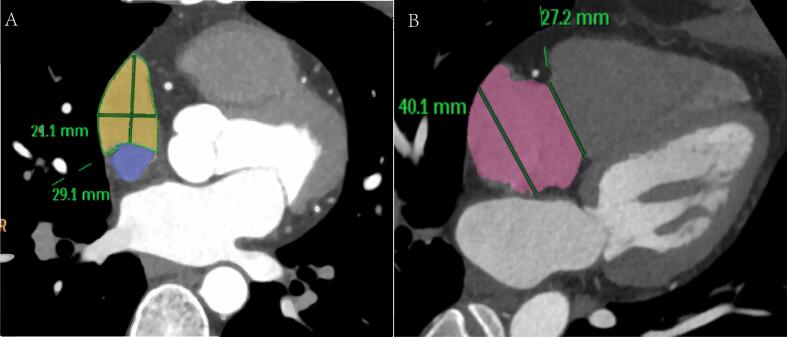
Measurement of the long and short diameters, circumference, and area of the RAA base on axial images (Fig. 2A). Measurement of the tricuspid annulus diameter and the RA anteroposterior diameter at the four-chamber heart level (Fig. 2B).

**Fig. 3:**
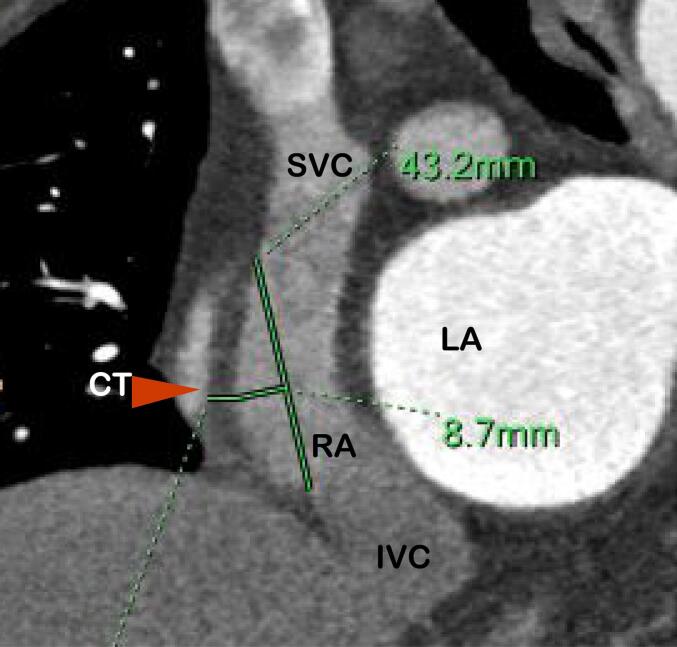
Measurement of the crista terminalis (CT)thickness: The optimal coronal image to display the crista terminalis was determined, which extends from the superior vena cava to the inferior vena cava. A line was drawn from the right margin of the superior vena cava (SVC) to the right margin of the inferior vena cava (IVC), and intersect this line with a perpendicular line passing through its midpoint. The thickness of the crista terminalis in this region was measured.

**Fig. 4:**
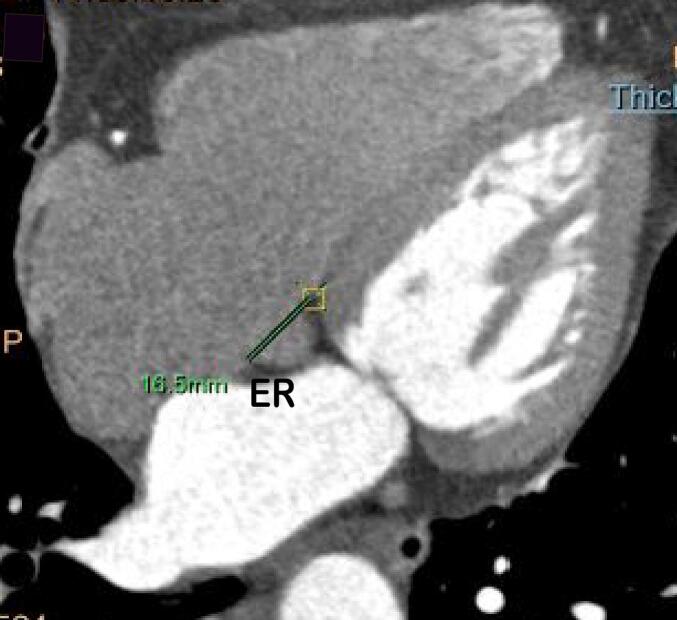
The length of the *para*-septal isthmus was measured as the distance between the attachment of the tricuspid septum and the Eustachian ridge (ER).

**Fig. 5:**
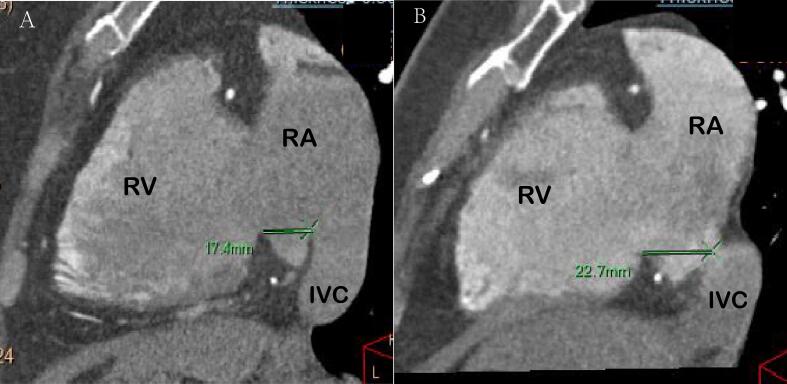
The lengths of the central (Fig. 5A) and lateral isthmus (Fig. 5B) were measured as the distance from the anterior and lateral edges of the IVC to the tricuspid annulus on the two-chamber plane of the right ventricle, respectively.

The measurement methods were described as follows. (1) Cardiac function analysis software was applied to reconstruct 3D images of the RAA, RA, and LA and calculate their volumes, The RAA height was defined as the vertical distance from the RAA apex to its basal plane (Fig. 1A-B). (2) The RAA base was identified on axial images at the orifice level of the superior vena cava, where the long diameter, short diameter, circumference and area of the RAA base were subsequently measured (Fig. 2A). (3) The anteroposterior diameter of the RA and tricuspid annulus diameter were measured at the four-chamber level [9], [10] (Fig. 2B). (4) Coronal images were adjusted to an optimal plane to visualize the crista terminalis extending from the SVC to the inferior vena cava (IVC). A straight line was drawn connecting the right margins of the superior and inferior vena cava, and a perpendicular line crossing the midpoint of the above line was marked to intersect the crista terminalis. The thickness of the crista terminalis at this intersection was then measured (Fig. 3) [11], [12]. [5] The cavo–tricuspid valve isthmus (CTI) consists of three components: the *para*-septal isthmus, central isthmus, and lateral isthmus [12], [13]. The *para*-septal isthmus was visualized on the four-chamber cardiac plane at the central level of the coronary sinus ostium. The length of the *para*-septal isthmus was defined as the distance between the septal attachment of the tricuspid valve and the Eustachian ridge (ER) at the ostium of the inferior vena cava (Fig. 4). For the central and lateral isthmuses, their lengths were measured respectively at the right ventricular two-chamber cardiac plane: specifically, the distance from the anterior edge of the inferior vena cava to the tricuspid valve annulus for the central isthmus, and the distance from the lateral edge of the inferior vena cava to the tricuspid valve annulus for the lateral isthmus (Fig. 5A–B). Post-processing and image analysis were performed by two experienced radiologists without knowledge of the patient's clinical data.

### 2.3 Statistical analysis

SPSS 25.0 statistical software was used for data analysis. The Shapiro-Wilk normality test was performed to determine whether the data conformed to normal distribution. The independent sample *t*-test was used to analyze the data between the two groups that obeyed normal distribution, expressed as mean ± standard deviation (SD), while the data that did not obey normal distribution were analyzed by the Wilcoxon rank sum test, expressed as median (interquartile range). The chi-square test was used to compare count data, expressed as percentages (%). Univariate and multivariate Logistic regression analysis was performed on the variables with statistically significant differences in the aforementioned tests to identify potential relevant factors associated with recurrence in male and female patients with AF. A multicollinearity test was conducted by calculating the variance inflation factor (VIF) for each variable to exclude variables with strong collinearity. Risk factors were used to build a risk prediction model, and receiver operating characteristic (ROC) curves were used to evaluate the accuracy of the prediction model. P value < 0.05 was considered statistically significant.

## 3 Results

### 3.1 Comparison of clinical data and morphological parameters of the RAA, RA, and related structures between male and female AF groups

Among the 321 patients, 184 were males with an average age of 60.37 ± 13.48 years, whereas 137 were females with an average age of 62.91 ± 9.79 years. Baseline clinical characteristics, including hypertension, coronary heart disease, diabetes mellitus, hyperlipidemia, stroke/transient ischemic attack (TIA), heart failure, persistent AF (PeAF), recurrence rate, AF duration, and body mass index, were comparable between male and female AF patients, with no significant intergroup differences (all P > 0.05; Table 1).The female AF group exhibited a significantly higher CHA_2_DS_2_-VASc score than the male group (P < 0.05). After normalization by body surface area (BSA), comparison of CT-derived parameters of the RAA and RA revealed that females had significantly larger RA volume/BSA and RA anteroposterior diameter/BSA than males (P < 0.05; Table 2).No significant BSA-normalized differences were observed between the two groups in RAA volume, RAA height; short diameter, long diameter, area, circumference of the RAA base; tricuspid annulus diameter, crista terminalis thickness, *para*-septal isthmus, central isthmus, lateral isthmus, and LA volume (all P > 0.05).

**Table 1:** Clinical characteristics.

Characteristics	Male(n = 184)	Female (n = 137)	P
Age (years)	60.37 ± 13.48	62.91 ± 9.79	0.051
Hypertension (%)	92(50.0%)	83(60.6%)	0.060
Coronary heart disease (%)	105(57.1%)	69(50.4%)	0.233
Diabetes (%)	26(14.1%)	28(20.4%)	0.135
Stroke/TIA (%)	34(18.5%)	23(16.8%)	0.695
Heart failure (%)	42(22.8%)	38(27.7%)	0.314
Hyperlipidemia (%)	35(19.0%)	36(26.3%)	0.121
PeAF(%)	46(25.0%)	34(24.8%)	0.970
Recurrence (%)	50(27.2%)	46(33.6%)	0.215
AF duration (months)	24(2,48)	15(4,48)	0.968
CHA2DS2-VASc score	2(1,3)	3(2,4)	0.000
BMI(kg/m^2^)	26.17 ± 4.15	25.44 ± 3.41	0.093

**Table 2:** Comparison of the CT-related parameters after BSA normalization

CT-related parameters	Male (n = 184)	Female (n = 137)	P
RAA volume /BSA(ml/m^2^)	5.95 ± 2.77	5.84 ± 2.58	0.700
RA volume /BSA(ml/m^2^)	44.10 ± 13.15	47.91 ± 15.91	0.023
LA volume /BSA(ml/m^2^)	55.93 ± 18.12	56.61 ± 17.53	0.737
Height of RAA /BSA(mm/m^2^)	15.12 ± 3.16	15.27 ± 2.84	0.814
RAA base			
Long diameter /BSA(mm/m^2^)	19.92 ± 3.77	20.38 ± 3.03	0.220
Short diameter /BSA(mm/m^2^)	13.43 ± 3.16	13.72 ± 3.55	0.443
circumference /BSA(mm/m^2^)	61.17 ± 9.90	62.86 ± 9.31	0.122
Area /BSA(mm^2^/m^2^)	420.45 ± 121.97	435.47 ± 128.26	0.287
RA anteroposterior diameter /BSA(mm/m^2^)	25.38 ± 3.79	26.37 ± 3.82	0.021
Tricuspid annulus diameter /BSA (mm/m^2^)	20.62 ± 3.31	21.09 ± 3.11	0.201
Para-septal isthmus /BSA(mm/m^2^)	8.84 ± 1.25	9.10 ± 1.24	0.064
Central isthmus /BSA(mm/m^2^)	11.23 ± 2.08	11.64 ± 2.38	0.096
Lateral isthmus /BSA(mm/m^2^)	13.19 ± 2.56	13.39 ± 2.34	0.486

### 3.2 Comparison of baseline characteristics and morphological parameters of RAA, RA and related structures between recurrent and non-recurrent patients in male and female groups

Male and female patients were further divided into recurrent and non– recurrent groups. Baseline data showed satisfactory intergroup balance, with trivial disparities limited to several non-primary indicators. Female patients with AF recurrence were more likely to have PeAF and heart failure, whereas male recurrent cases had higher proportion of PeAF, higher CHA_2_DS_2_-VASc scores and longer AF duration. In both male and female groups, recurrent cases exhibited significantly larger volumes of the RA, RAA, and LA; as well as greater RAA height, short diameter, area and circumference of RAA base; RA anteroposterior diameter, tricuspid annulus diameter, *para*-septal isthmus, central isthmus and lateral isthmus, crista terminalis thickness, right atrial volume index (RAVI), and left atrial volume index (LAVI) (P < 0.05). In female patients alone, the long diameter of the RAA base was also significantly increased in the recurrent subgroup (P < 0.05; Table 3, Table 4).

**Table 3:** Comparison of baseline characteristics and parameters of RAA, RA, and related structures in Female recurrent patients and non-recurrent patients.

Characteristics	AF recurrence(n = 46)	AF non-recurrence(n = 91)	P
Age (years)	60.80 ± 11.35	63.98 ± 8.77	0.101
Hypertension (%)	24(52.2%)	59(64.8%)	0.152
Coronary heart disease (%)	19(41.3%)	50(54.9%)	0.132
Diabetes (%)	12(26.1%)	16(17.6%)	0.267
Stroke/TIA (%)	5(10.9%)	18(19.8%)	0.188
Heart failure (%)	22(47.8%)	16(17.6%)	0.000
Hyperlipidemia (%)	11(23.9%)	25(27.5%)	0.655
PeAF (%)	23(50.0%)	11(12.1%)	0.000
AF duration (months)	24(4,72)	12(3,48)	0.081
CHA2DS2-VASc score	4(2,4)	3(2,4)	0.387
BMI(kg/m^2^)	25.45 ± 3.63	25.44 ± 3.31	0.994
CT-related parameters			
RAA volume (mL)	13.10 ± 5.31	8.33 ± 3.54	0.000
RA volume (mL)	91.85 ± 34.62	72.16 ± 17.52	0.001
LA volume (mL)	117.27 ± 38.11	93.66 ± 21.61	0.010
Height of RAA (mm)	28.47 ± 4.40	24.58 ± 4.25	0.000
RAA base			
Long diameter (mm)	37.22 ± 5.87	34.17 ± 5.17	0.002
Short diameter (mm)	28.80 ± 4.92	22.52 ± 5.13	0.000
circumference (mm)	119.17 ± 15.63	105.05 ± 13.16	0.000
Area (mm^2^)	924.61 ± 246.80	684.23 ± 175.09	0.000
RA anteroposterior diameter (mm)	48.08 ± 6.88	44.06 ± 6.29	0.001
Tricuspid annulus Diameter (mm)	37.21 ± 4.20	34.89 ± 4.80	0.006
Crista terminalis thickness (mm)	3.75 ± 0.72	3.34 ± 0.59	0.000
Para-septal isthmus (mm)	16.19 ± 1.89	15.57 ± 1.33	0.030
Central isthmus (mm)	21.81 ± 3.51	19.29 ± 2.92	0.000
Lateral isthmus (mm)	25.75 ± 3.88	23.28 ± 3.19	0.000
RAVI (mL/m2)	54.69 ± 20.02	43.62 ± 10.78	0.001
LAVI (mL/m2)	69.94 ± 22.12	56.63 ± 13.30	0.000

**Table 4:** Comparison of baseline characteristics and parameters of RAA, RA, and related structures in Male recurrent patients and non-recurrent patients.

Characteristics	AF recurrence(n = 50)	AF non-recurrence(n = 134)	P
Age (years)	61.24 ± 14.01	60.04 ± 13.32	0.594
Hypertension (%)	29(58.0%)	63(47.0%)	0.185
Coronary heart disease (%)	33(66.0%)	72(53.7%)	0.233
Diabetes (%)	11(22.0%)	15(11.2%)	0.061
Stroke/TIA (%)	10(20.0%)	24(17.9%)	0.695
Heart failure (%)	15(30.0%)	27(20.1%)	0.157
Hyperlipidemia (%)	11(22.0%)	24(17.9%)	0.121
PeAF(%)	20(40.0%)	26(19.4%)	0.004
AF duration (months)	36(6,84)	12(2,36)	0.005
CHA2DS2-VASc score	3(1,3)	2(1,3)	0.027
BMI(kg/m^2^)	25.75 ± 3.04	26.33 ± 4.50	0.394
CT-related parameters			
RAA volume (mL)	14.76 ± 6.23	9.83 ± 4.25	0.000
RA volume (mL)	95.07 ± 33.32	79.89 ± 21.82	0.004
LA volume (mL)	116.17 ± 40.82	93.03 ± 27.40	0.000
Height of RAA (mm)	31.16 ± 5.35	26.92 ± 5.33	0.000
RAA base			
Long diameter (mm)	36.82 ± 5.33	36.02 ± 5.69	0.392
Short diameter (mm)	28.27 ± 5.71	22.22 ± 5.21	0.000
circumference (mm)	118.75 ± 15.70	107.32 ± 14.02	0.000
Area (mm^2^)	901.75 ± 252.88	710.28 ± 197.13	0.000
RA anteroposterior diameter (mm)	48.81 ± 6.55	45.44 ± 5.20	0.000
Tricuspid annulus Diameter (mm)	40.47 ± 5.22	37.32 ± 5.77	0.001
Crista terminalis thickness (mm)	4.12 ± 0.87	3.71 ± 0.72	0.002
Para-septal isthmus (mm)	16.56 ± 2.18	15.79 ± 1.60	0.025
Central isthmus (mm)	21.76 ± 3.73	19.23 ± 3.01	0.000
Lateral isthmus (mm)	25.89 ± 5.16	23.00 ± 3.61	0.000
RAVI (mL/m2)	50.21 ± 17.87	42.40 ± 11.13	0.005
LAVI (mL/m2)	61.28 ± 21.06	49.34 ± 13.97	0.000

### 3.3 Univariate and multivariate logistic regression analyses

Univariate logistic regression analysis revealed that the RAA volume, RA volume, RAA height; short diameter, circumference, and area of the RAA base; RA anteroposterior diameter, tricuspid annulus diameter, RAVI, LAVI, LA volume, crista terminalis thickness, *para*-septal isthmus, central isthmus, lateral isthmus, and persistent AF were significant predictors of AF recurrence after RFA in both female and male groups (P < 0.05). In the female group, the long diameter of the RAA base and heart failure were identified as significant predictors of AF recurrence after RFA (P < 0.05). In the male group, the CHA2DS2-VASc score and AF duration were significant predictors of AF recurrence after RFA (P < 0.05; Table 5, Table 6). Variables with a VIF greater than 10 were as follows: in the female group, the area of the RAA base (30.697), RAVI (506.822), LA volume (15.858), RAA volume (11.261), circumference of the RAA base (10.928); in the male group, the area of the RAA base (33.360), RAVI (299.896), and LA volume (363.761). These variables were excluded due to the presence of multicollinearity. All other variables had a VIF of less than 10, which were eligible for inclusion in the multivariate logistic regression analysis.

**Table 5:** Logistic regression analysis of variables with Female recurrence.

Variables	Univariate analysis OR (95% CI) *p-*value		Multivariate analysis OR (95% CI) *p-*value	
RAA volume	1.299 (1.166–1.447)	0.000		
RA volume	1.032 (1.015–1.049)	0.000	0.995 (0.960–1.031)	0.779
LA volume	1.031 (1.015–1.046)	0.000		
RAA height	1.226 (1.117–1.346)	0.000	1.116 (0.984–1.266)	0.088
Long diameter of RAA base	1.108 (1.034–1.187)	0.004	0.987 (0.871–1.119)	0.836
Short diameter of RAA base	1.271 (1.161–1.391)	0.001	1.227(1.069–1.408)	0.004
Circumference of RAA base	1.071 (1.040–1.104)	0.001		
Area of RAA base	1.005 (1.003–1.008)	0.000		
RA anteroposterior diameter	1.117 (1.045–1.194)	0.025	0.965 (0.882–1.056)	0.441
Tricuspid annulus diameter	1.132 (1.034–1.240)	0.007	1.025 (0.877–1.199)	0.754
Crista terminalis thickness	2.625 (1.466–4.701)	0.001	2.493 (1.123–5.535)	0.025
Para-septal isthmus	1.302 (1.016–1.668)	0.037	1.100 (0.785–1.540)	0.581
Central isthmus	1.284 (1.133–1.456)	0.000	1.137(0.840–1.539)	0.406
Lateral isthmus	1.233 (1.099–1.383)	0.000	0.879 (0.662–1.168)	0.375
RAVI	1.052 (1.023–1.082)	0.000		
LAVI	1.050(1.023–1.077)	0.003	1.023 (0.982–1.065)	0.280
PeAF	7.273(3.093–17.103)	0.000	4.211(1.092–16.238)	0.037
Heart failure	4.297 (1.948–9.479)	0.000	1.652(0.508–5.371)	0.404

**Table 6:** Logistic regression analysis of variables with Male recurrence.

Variables	Univariate analysis OR (95% CI) *p-*value		Multivariate analysis OR (95% CI) *p-*value	
RAA volume	1.203 (1.115–1.298)	0.000	0.945 (0.772–1.156)	0.580
RA volume	1.022 (1.009–1.036)	0.001	1.015 (0.987–1.042)	0.295
LA volume	1.022 (1.011–1.033)	0.000		
RAA height	1.146 (1.077–1.221)	0.001	1.089 (0.974–1.218)	0.133
Short diameter of RAA base	1.217 (1.135–1.306)	0.000	1.226 (1.063–1.413)	0.005
Circumference of RAA base	1.054 (1.029–1.080)	0.000	0.971(0.917–1.028)	0.307
Area of RAA base	1.004 (1.002–1.005)	0.000		
RA anteroposterior diameter	1.115 (1.046–1.187)	0.001	0.932 (0.823–1.054)	0.261
Tricuspid annulus diameter	1.104 (1.038–1.173)	0.002	1.044 (0.933–1.167)	0.453
Crista terminalis thickness	1.909 (1.252–2.991)	0.003	1.656 (0.983–2.788)	0.058
Para-septal isthmus	1.264 (1.054–1.516)	0.011	1.063(0.805–1.404)	0.667
Central isthmus	1.252 (1.129–1.388)	0.000	1.123(0.911–1.384)	0.279
Lateral isthmus	1.180 (1.085–1.283)	0.000	0.988 (0.822–1.188)	0.901
RAVI	1.042 (1.017–1.068)	0.001		
LAVI	1.043(1.022–1.065)	0.000	1.010 (0.973–1.048)	0.604
CHA2DS2-VASc score	1.321(1.059–1.647)	0.013	1.273 (0.938–1.729)	0.121
PeAF	2.769(1.362–5.630)	0.005	1.123(0.414–3.043)	0.820
AF duration	1.008 (1.002–1.014)	0.011	1.009 (1.002–1.017)	0.013

Multivariate logistic regression analysis demonstrated that the short diameter of the RAA base, crista terminalis thickness, and persistent AF were independent predictors of post- RFA recurrence in female patients with AF (Table 5; P < 0.05). Furthermore, in male patients with AF, the short diameter of the RAA base and AF duration were independent predictors of recurrence after RFA (Table 6; p < 0.05).

### 3.4 Parameter prediction values

**Fig. 6:**
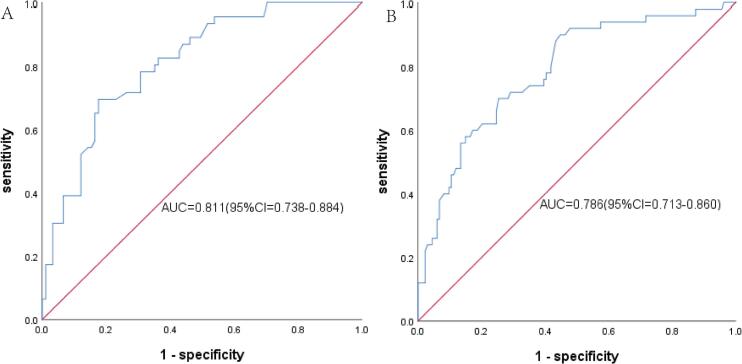
ROC curve of multiple logistic regression analysis were drawn for the short diameter of the RAA base in female patients (Fig. 6A) and male patients (Fig. 6B).

ROC curves were constructed for the short diameter of the RAA base, crista terminalis thickness, and persistent AF in female patients, as well as for the short diameter of the RAA base and AF duration in male patients. In female patients, the short diameter of the RAA base > 26.95 mm exhibited the highest predictive value for AF recurrence, with a sensitivity of 0.696, a specificity of 0.824, and an area under the curve (AUC) of 0.811 (P = 0.000) (Fig. 6A). The crista terminalis thickness > 3.25 mm had predictive value (AUC = 0.689, P = 0.000; sensitivity = 0.783, specificity = 0.549). Persistent AF also exhibited predictive significance (AUC = 0.690, P = 0.000). In male patients, the short diameter of the RAA base > 25.55 mm showed the highest predictive value for AF recurrence, with a sensitivity of 0.700, a specificity of 0.746, and an AUC of 0.786 (P = 0.000) (Fig. 6B). AF duration > 44 months also predicted AF recurrence (AUC = 0.633, sensitivity 0.460, specificity 0.769; P = 0.006).

## 4 Discussion

AF is the most prevalent cardiac arrhythmia. Previous population cohort studies have confirmed that there are significant gender differences in the prognosis of AF [14]. Some scholars have explored gender-related differences in LA substrate using high-precision mapping techniques [5], and found that compared with male patients, female AF patients have lower LA voltage, slower electrical conduction velocity, and more complex abnormal potentials. These electrophysiological characteristics may be important mechanisms contributing to the higher recurrence risk of female AF patients after catheter ablation. Another study suggested that when the CHA_2_DS_2_-VASc score is ≥ 3, the proportion of LA low-voltage areas in female patients is significantly higher than that in male patients [15]. In our study, patients were grouped by gender. Although the difference in recurrence rate between the two groups did not reach statistical significance, a higher post-operative recurrence rate was still observed in female AF patients compared with male patients. Meanwhile, the CHA_2_DS_2_-VASc score of female patients was significantly higher than that of male patients, and the difference was statistically significant (P < 0.05). Therefore, clinical practice should formulate more refined pre-operative risk assessment and individualized long-term post-operative management strategies for female AF patients, so as to improve their surgical benefits and long-term prognosis.

Catheter ablation for AF has become the first-line treatment option for drug-refractory AF [16], but the postoperative recurrence rate can be as high as 10%–30% (1). The relatively high recurrence after ablation affects its clinical application and treatment effect to some extent. Therefore, it is crucial to effectively identify the risk factors for AF recurrence following RFA. Akutsu Y et al. [17] demonstrated that RA remodeling was associated with AF recurrence following RFA, and their findings identified larger RA volume and LA volume as independent predictors of AF recurrence after ablation. This study evaluated the relationship between RAA- and RA-related anatomical structures and postoperative ablation in different genders. The results showed that structural parameters of RAA and RA increased in both male and female patients with AF recurrence after surgery. Thus, both male and female AF patients with larger anatomical diameters of the RAA and RA have a higher risk of postoperative recurrence, which is consistent with previous studies [18], and can provide a certain reference value for RFA due to AF.ROC curves indicated that the short diameter of the RAA base had high accuracy in predicting postoperative recurrence in both male and female AF patients. Specifically, female AF patients with the short diameter of RAA base > 26.95 mm and male AF patients with the short diameter of RAA base > 25.55 mm were more likely to experience postoperative recurrence. The underlying mechanism may be that the increased short diameter of the RAA base can induce myocardial interstitial fibrosis, endocardial remodeling, and cardiomyocyte hypertrophy, leading to electrophysiological alterations of ion channels, which in turn enhance myocardial excitability and automaticity and thereby trigger AF. Therefore, the increased short diameter of the RAA base is associated with a lower rate of postoperative sinus rhythm maintenance.

The RA and RAA remain understudied cardiac chambers. Previous studies have generally acknowledged that the LA plays a dominant role in the pathophysiological progression of AF [19]. With the rapid advances in interventional cardiology, a thorough understanding of the anatomical characteristics of the RA and RAA is of great guiding significance for the clinical management of primary RA electrical disorders and the optimization of catheter ablation strategies for AF patients (9). Currently, comparative studies exploring gender-based differences in RA and RAA anatomical structures among AF patients are still scarce. Given the inherent physical disparity between male and female patients, normalization of RA and RAA measurements by BSA can eliminate confounding effects of body size, enable objective comparisons among individuals with different physiques, and more accurately reflect the actual anatomical alterations. In the present study, we analyzed gender differences in RA and RAA structural parameters in AF patients. The results revealed that BSA-indexed RA volume and anteroposterior diameter were significantly larger in female AF patients than in male counterparts, with statistically significant differences (P < 0.05). These findings indicate that after adjusting for body size, female AF patients exhibit more prominent RA structural remodeling characterized by enlarged RA volume and increased RA anteroposterior diameter compared with male patients.

Understanding the anatomical structures of the RA and RAA is conducive to improving the safety of AF-related interventional and electrophysiological procedures and shortening the operation time. Transesophageal echocardiography (TEE), featuring low cost and real-time imaging, is the preferred method for evaluating right heart structure before and during surgery [12]. However, this examination is semi-invasive, prone to causing patient discomfort, and its manual measurement is highly subjective with limited accuracy. Cardiac magnetic resonance (CMR) is the gold standard for cardiac chamber volume assessment, which is free of ionizing radiation and can accurately evaluate myocardial fibrosis [20]. Nevertheless, it has low spatial resolution and long scanning time, making it unsuitable for patients with claustrophobia or pacemaker implantation. CCTA has excellent spatial resolution and can clearly evaluate the anatomical morphology of the right heart [12], [21]. When combined with a dual-tube high-pressure injector (Stellant) and contrast injection software (P3T software) [22], it can further optimize imaging quality and ensure measurement accuracy. Although there is an excellent correlation between CCTA and CMR measurement results [23], CCTA carries risks of ionizing radiation and iodine contrast agent allergy. The aforementioned cardiac imaging techniques have enriched the anatomical details of the RAA and RA, providing sufficient and detailed anatomical evidence for clinical research and preoperative evaluation [12], [24].

## 5 Limitations

This was a single-center retrospective study with several limitations. First, the relatively short follow-up duration may lead to underestimation of AF recurrence rates after ablation; furthermore, the absence of an external validation cohort limits the generalizability of the conclusions and carries a risk of overfitting. Second, although variables were selected using multicollinearity analysis, the model still partially relies on imaging parameters with strong correlations. Finally, complete invasive voltage mapping and Late Gadolinium Enhancement-CMR data were unavailable in this study, which restricted direct correlation analysis and hindered the elucidation of the underlying pathophysiological mechanisms.

Future research directions include expanding the sample size for external validation, conducting prospective studies, integrating multimodal data, and establishing more robust prediction models using machine learning. Further studies will also be performed to investigate in depth the relationships between structural parameters of the RAA and RA with atrial fibrosis and electrophysiological substrate, as well as to analyze the mechanisms underlying gender differences.

## 6 Conclusions

This study quantitatively analyzed the associations between anatomical parameters of the RAA and RA and postoperative recurrence after radiofrequency ablation in AF patients of different genders. Among all indicators, the short diameter of the RAA base exhibited the highest predictive value for postoperative AF recurrence in both male and female patients. Collectively, our findings can effectively predict postoperative recurrence after RFA for AF, and can provide imaging reference basis for preoperative risk stratification and individualized treatment planning.

## CRediT authorship contribution statement

**Tong Pan:** Writing – review & editing, Writing – original draft, Methodology, Investigation, Formal analysis, Data curation. **Yang Liu:** Data curation. **Cai-Ying Li:** Writing – review & editing. **Shi-Bo Dong:** Data curation. **Dan Zhang:** Data curation. **Xiao-Nan Han:** Data curation.

## Funding

The authors declare that no funds, grants, or other support were received during the preparation of this manuscript.

## Declaration of competing interest

The authors declare that they have no known competing financial interests or personal relationships that could have appeared to influence the work reported in this paper.
